# The Pepper E3 Ligase CaGIR1 Acts as a Negative Regulator of Drought Response via Controlling CaGRAS1 Stability

**DOI:** 10.1111/pce.15516

**Published:** 2025-04-08

**Authors:** Woonhee Baek, Donghyuk Oh, Lim Chae Woo, Sung Chul Lee

**Affiliations:** ^1^ Department of Life Science (BK21 Programme) Chung‐Ang University Seoul South Korea

**Keywords:** drought stress, E3 ligase, GRAS transcription factor, pepper, ubiquitination

## Abstract

The ubiquitin‐proteasome pathway modulates protein stability, which impacts plant responses to abiotic stresses, such as drought. Our previous study reported that the pepper GRAS‐type transcription factor CaGRAS1 plays a positive role in drought resistance. However, the mechanism by which drought stress affects CaGRAS1 protein stability remains unknown. Here, we identified *Capsicum annuum CaGRAS1*‐Interacting RING‐type E3 ligase 1 (CaGIR1) through yeast two‐hybrid analysis. The interaction between these two proteins was confirmed by both in vitro and in vivo assays, and interaction occurred in both the nucleus and cytoplasm, consistent with their subcellular localisation. In ubiquitination assays, CaGIR1 was shown to have ubiquitin E3 ligase activity, which is dependent on its RING domain. CaGIR1 also directly ubiquitinated CaGRAS1 in vitro and in vivo, and CaGRAS1 protein stability negatively correlated with *CaGIR1* expression levels. In contrast to CaGRAS1, CaGIR1 was found to play a negative role in drought resistance. Phenotypic assays revealed that the silencing of *CaGIR1* in pepper resulted in enhanced drought resistance through the modulation of stomatal responses and drought‐responsive marker gene expression, whereas *CaGIR1* overexpression led to the opposite results in Arabidopsis. Overall, our findings suggest that CaGIR1 negatively modulates ABA and drought responses by triggering CaGRAS1 protein degradation.

## Summary statement

Under drought stress, plants defend themselves by activating stress‐related transcription factors, which regulate protein stability or activity in response to surrounding environmental conditions. In this study, the drought‐inducible pepper RING‐type E3 ligase CaGIR1 negatively regulated drought responses by modulating the protein stability of CaGRAS1, which was previously reported to positively regulate the ABA‐mediated drought response in pepper. Our results suggest that the new regulatory mechanism in E3 ligase—GRAS family transcription factor involved in drought resistance response.

## 1 Introduction

Because water is the most critical ingredient in a plant's life, water deficiency impairs normal growth, interferes with nutrient transport, and significantly reduces the efficiency of water use in plants (Colin et al. 2022). To withstand these unfavourable changes as sessile organisms, plants have developed adaptive mechanisms for minimising water loss from shoots and maximising water uptake through their roots (Hu and Xiong 2014). In response to drought, abscisic acid (ABA) accumulates in various plant tissues, especially the vasculature of leaves (Raghavendra et al. 2010; Kuromori et al. 2018). This well‐known plant stress hormone plays a crucial role in a variety of stress responses, including stomatal closure and the expression of stress‐responsive genes (Cutler et al. 2010). In the steps from ABA perception to downstream responses, three core components of ABA signalling are involved: the soluble ABA receptors PYR/PYL/RCAR (PYRABACTIN RESISTANCE/PYRABACTIN RESISTANCE‐LIKE/REGULATORY COMPONENT OF ABA RECEPTOR), CLADE A PROTEIN PHOSPHATASE 2Cs (PP2Cs; negative regulators), and SNF1 (sucrose non‐fermenting1)‐related kinases (SnRKs) (Fujita et al. 2009; Ma et al. 2009; Nakashima et al. 2009; Park et al. 2009; Cutler et al. 2010). When ABA enters into the plant cell, PYR/PYL/RCARs bind to ABA and help to form a trimeric RCAR–ABA–PP2C complex, which relieves the PP2C‐mediated inhibition of SnRK2; in turn, the released SnRK2s modulate the activity of various downstream targets, such as transcription factors and ion channels (Lee et al. 2009; Sato et al. 2009; Umezawa et al. 2009; Ng et al. 2011; Santiago et al. 2012; Soon et al. 2012; Yoshida et al. 2015).

Plant transcription factors play a pivotal role in the regulation of gene expression in response to stress and hormones. Many studies have revealed that transcription factor activity is influenced by post‐translational modifications (PTMs), such as phosphorylation/dephosphorylation, sumoylation, and ubiquitination (Downes and Vierstra 2005; Hunter 2009; Joo 2021). As an example, downstream transcription factors involved in ABA signalling, such as ABA‐INSENSITIVE5 (ABI5) and ABA‐RESPONSIVE ELEMENTS BINDING FACTORS (ABFs), have been known to be regulated by various PTMs (Yang et al. 2017; Zhang et al. 2019). One of the most common PTMs is ubiquitination that entails the attachment of ubiquitin to lysine residues on target proteins. Ubiquitin (Ub) proteins are characterised by seven lysine residues, consistently located at positions K6, K11, K27, K29, K31, K48, and K63, without exception. Particularly, K48‐linked polyubiquitin (polyUb) chains are the most abundant in Arabidopsis and serve as a canonical signal for the degradation of ubiquitinated proteins through the 26S proteasome system (Maor et al. 2007; Sadanandom et al. 2012; Callis 2014). Genomic analyses demonstrate that core components related to ubiquitination machinery comprise approximately 6% of the Arabidopsis (*Arabidopsis thaliana*) proteome (Vierstra 2009; Skelly et al. 2016). The ubiquitination pathway consists of three enzymes—ubiquitin‐activating enzyme (E1), ubiquitin‐conjugating enzyme (E2), and E3 ubiquitin ligase—all of which sequentially catalyse the attachment of ubiquitin to substrates (Moon et al. 2004; Stone 2014). In particular, E3 ubiquitin ligase determines substrate specificity through interactions with substrates (Stone 2019). Over the past two decades, many E3 ligases have been found to interact with substrate proteins, including transcription factors, involved in ABA signalling and drought stress responses (Yang et al. 2017). These E3 ligase‐mediated regulations have both positive and negative impacts on ABA signalling and drought stress responses. As a negative regulator of ABA signalling, RING‐type E3 ligase KEG (KEEP ON GOING) interacts with ABI5 (Liu and Stone 2010) and ABF1/3 (Chen et al. 2013), and affects their protein stability. ABI5 is also found to interact with other types of E3 ligases, such as CUL‐4‐DDB‐type DWA1/2 (Lee et al. 2010) and ABD1 complexes (Seo et al. 2014). In addition, SDIR1 directly interacts with and ubiquitinates SDIRIP1 (SDIR1‐INTERACTING PROTEIN 1), which selectively regulates *ABI5* gene expression during ABA signalling (Zhang et al. 2015). The RING‐type E3 ligases CaDSR1 and CaASRF1/CaATIR1 are reported to be responsible for ubiquitinating the bZIP transcription factors CaDILZ1 and CaAIBZ/CaATBZ1, which contribute positively and negatively, respectively, to drought resistance in pepper plants (Lim et al. 2018; Joo et al. 2019; Joo et al. 2020). In gibberellin signalling, GRAS transcription factor DELLA proteins have been shown to be regulated by the SCF^SLY1/GID2^ ubiquitin E3 ligase complex (Dill et al. 2004; Fu et al. 2004) and COP1 E3 ligase (Blanco‐Tourinan et al. 2020). These reports support the notion that ubiquitination‐mediated regulation of transcription factors is essential for plant adaptation to a changing environment.

Previously, we isolated the drought‐induced pepper GRAS‐type transcription factor CaGRAS1, which plays a role as a positive regulator in ABA‐mediated drought stress response (Oh et al. 2022). To better understand the biological function of CaGRAS1, we searched for proteins that can interact with CaGRAS1 and influence its activity, abundance, or stability. Here, we isolated a RING‐type E3 ligase that interacted with CaGRAS1 by yeast two‐hybrid assay and named it CaGIR1 (*Capsicum annuum* CaGRAS1 Interacting RING‐type E3 ligase 1). In vivo and in vitro ubiquitination assays revealed that the CaGIR1 protein possesses E3 ligase activity and ubiquitinates CaGRAS1, which contributes to the degradation of CaGRAS1 proteins under drought stress conditions. In phenotypic analyses, virus‐induced silencing of the *CaGIR1* gene enhanced drought resistance in pepper plants, whereas overexpression of *CaGIR1* reduced drought resistance in Arabidopsis. Our study demonstrates that the CaGIR1 protein plays a negative role in ABA signalling and drought response, and in the process modulates CaGRAS1 stability.

## 2 Materials and Methods

### 2.1 Plant Materials and Growth Conditions

Seeds of hot pepper (*Capsicum annuum* L. cv. Nockwang) and tobacco (*Nicotiana benthamiana*) were plated on wet paper towels at 28°C. After germination, 7‐day‐old seedlings of pepper and tobacco plants were transplanted and grown in a commercial soil mixture (Sunshine Mix #5, Sun Grow Horticulture) at 25°C under a long‐day photoperiod (16‐h light/8‐h dark). *Arabidopsis thaliana* (Col‐0 ecotype) seeds were surface sterilised with 10% NaOCl for 20 min and plated on half‐strength MS medium with 1% sucrose and 0.8% agar. To select transgenic plants, we added antibiotics as necessary. Following stratification for 2 days at 4°C, Arabidopsis seeds were grown under a long‐day photoperiod in a growth room at 23°C.

### 2.2 Virus‐Induced Gene Silencing (VIGS)

*CaGIR1*‐silenced pepper plants were generated using the tobacco rattle virus (TRV)‐based VIGS system as described previously (Liu et al. 2002; Oh et al. 2022). A target region for gene silencing in pepper plants was determined using the VIGS tool on the Sol Genomics Network (https://vigs.solgenomics.net/). A 265‐bp fragment of the *CaGIR1* gene was then inserted into the pTRV2 vector and digested by *Xba*I and *Xho*I. *Agrobacterium tumefaciens* containing pTRV2:*CaGIR1* and pTRV1 (OD_600_ = 0.2 for each construct) were co‐infiltrated into fully expanded cotyledons from pepper plants using a needleless syringe. pTRV2:00 was used as a negative control.

### 2.3 Generation of *CaGIR1*‐Overexpression Arabidopsis Plants

To generate the *CaGIR1*‐overexpression Arabidopsis plants, we amplified the CaGIR1 coding sequence (CA09g13790) using cDNA from pepper leaves and cloned this sequence into a pCR™8/GW/TOPO™ vector (Thermo Fisher Scientific, Waltham, MA, USA). Through the LR reaction, *Pro35S‐CaGIR1* constructs were produced and then transformed into *A. tumefaciens*. Following floral dip transformation (Clough and Bent 1998), *Pro35S‐CaGIR1* Arabidopsis mutants were initially selected on MS agar medium containing 25 μg mL^−1^ of phosphinothricin. The expression levels of *CaGIR1* in mutant plants were confirmed by PCR analysis with gene‐specific primers (Supporting Information S1: Table S1), and two independent T3 plant lines were used in this study.

### 2.4 Yeast Two‐Hybrid Assay

A yeast two‐hybrid assay was conducted as previously described (Lim et al. 2018). Briefly, the cDNA fragment of *CaGRAS1* (319–1749 bp) was used as bait because its N‐terminal region has auto‐activity in yeast. *CaGRAS1* and *CaGIR1* were inserted into pGBKT7 and pGADT7 vectors, respectively, and then were co‐transformed into yeast strain AH109 cells using the lithium acetate method. To verify the interaction between proteins, yeast transformants were grown on selection medium (SC adenine‐histidine‐leucine‐tryptophan [‐AHLW] medium).

### 2.5 Subcellular Localisation and Bimolecular Fluorescence Complementation (BiFC) Assay

To analyze the subcellular localisation of CaGIR1, we inserted the *CaGIR1* coding region lacking a stop codon into the green fluorescent protein (GFP)‐fused binary vector via the LR reaction. For agroinfiltration, *A. tumefaciens* GV3101 harbouring the *Pro35S:CaGIR1‐GFP* construct was mixed with the p19 strain (1:1 ratio; OD_600_ = 0.5) to avoid gene silencing, and the mixtures were infiltrated into *N. benthamiana* leaves using a needleless syringe. After 2 days, GFP signals were observed by confocal microscopy (Zeiss 710 UV/Vis Meta; Oberkochen, Germany) with LSM Image Browser software.

For the BiFC analysis, *CaGRAS1* and *CaGIR1* coding sequences were cloned into the Pro35S‐VYNE and Pro35S‐CYCE vectors, respectively. Transient gene expression was induced in *N. benthamiana* leaves by agroinfiltration as described above.

### 2.6 Luciferase Complementary Interaction Assay

The luciferase complementary interaction assay was performed according to a previous report with some modifications (Gehl et al. 2011). Full‐length cDNA of *CaGRAS1* and *CaGIR1* was cloned into the pDEST14‐N‐LUC vector (including an N‐terminal luciferase fragment) and the pDEST14‐C‐LUC vector (fused with a C‐terminal luciferase fragment). An *A. tumefaciens* GV3101 strain containing these constructs along with p19 strain cells were infiltrated into *N. benthamiana* leaves. After 3 days, inoculated leaf samples were infiltrated with 0.1 mM d‐luciferin (BioVision, Milpitas, CA, USA) and incubated in the dark for 30 min. Luciferase luminescence was observed using NightShade LB985 (Berthold Technologies, Bad Wildbad, Germany).

### 2.7 RNA Extraction and Quantitative Reverse Transcription‐Polymerase Chain Reaction (qRT‐PCR)

Total RNA was isolated from the leaves of pepper and Arabidopsis plants using an RNeasy Mini kit (Qiagen, Valencia, CA, USA) according to the manufacturer's instructions. Six‐leaf‐stage pepper plants were treated with drought stress by withholding watering, irrigating with a solution of 250 mM NaCl, and spraying plants with ABA (100 μM) or H_2_O_2_ (100 μM) solution as previously described (Lim et al. 2022; Oh et al. 2022). Leaves were then harvested at the indicated time points. Three‐week‐old Arabidopsis plants were subjected to drought stress in a similar manner. RNA samples were subjected to cDNA synthesis with Transcript First Strand cDNAs synthesis kits (Roche, Indianapolis, IN, USA). Quantitative RT‐PCR (qRT‐PCR) analysis was conducted using the iQ SYBR Green supermix with gene‐specific primers (Supporting Information S1: Table 1). The pepper *Actin1* gene (*CaACT1*) and Arabidopsis *Actin8* gene (*AtACT8*) were used as internal controls for normalisation.

### 2.8 Drought Resistance Assay

For the drought resistance assay, 4‐week‐old pepper plants (TRV2:*CaGIR1* and TRV2:00) and 3‐week‐old Arabidopsis plants (WT and *Pro35S:CaGIR1*) were exposed to drought stress by withholding watering for 12 days. Following 2–3 days of rehydration, the survival rates of each plant were assessed. To quantify leaf transpirational water loss, we detached leaves from 4‐week‐old pepper and 3‐week‐old Arabidopsis plants and dried these leaves in a growth chamber at 40% relative humidity. At the indicated time points, leaf weights were measured and transpirational water loss was calculated by comparing these weights with the initial weights.

### 2.9 Thermal Image Analysis

Thermal image analysis was conducted as previously described (Lim et al. 2018; Oh et al. 2022). To induce drought stress response, we treated 2‐week‐old pepper plants and 3‐week‐old Arabidopsis plants by withholding watering for 6–7 days. After treatment, thermal images were taken using an infrared camera (FLIR systems; T420). Leaf surface temperatures were measured from fully expanded first and second leaves of pepper plants and rosette leaves of Arabidopsis plants using FLIR Tools+ ver 5.2 software.

### 2.10 Stomatal Aperture Assay

For stomatal aperture assay, 3‐week‐old pepper or Arabidopsis plants were treated with drought by withholding watering for 6 days, and leaf epidermal peels were then collected. Approximately 130 stomata were randomly selected from each plant line, and images were captured using a Nikon Eclipse 80i fluorescence microscope. Pore width and length were measured using ImageJ software. Stomatal opening levels were classified based on the ratio of stomatal width to length: open (≥ 0.2), slightly open (0.05–0.2), closed (< 0.05), and fully closed, where guard cells were compressed.

### 2.11 Recombinant Protein Expression and Pull‐Down Assay

The recombinant proteins glutathione S‐transferase (GST)‐tagged CaGRAS1, and maltose binding protein (MBP)‐tagged CaGIR1 were expressed in bacterial cells and purified as described previously (Joo et al. 2019; Lim et al. 2022). Briefly, the full‐length coding sequences of the *CaGRAS1* and *CaGIR1* genes were cloned into pGEX4T‐3 (GE Healthcare Bio‐Sciences, Uppsala, Sweden) and pMAL‐c2X (New England Biolabs, Ipswich, MA, USA), respectively. Following induction with isopropyl β‐D‐1‐thiogalactopyranoside (ITPG), recombinant proteins were purified by glutathione Sepharose 4 fast flow (GE Healthcare Bio‐Sciences) and amylose resin (New England Biolabs) according to the manufacturers’ procedures. For the in vitro pull‐down assay, GST‐CaGRAS1 pre‐bounded with Sepharose resin was incubated with MBP‐CaGIR1 and MBP proteins from the *E. coli* (BL21) lysate, with continuous rotation for 2 h at 4°C and washed in STE buffer including 0.05% Triton X‐100, five times. The precipitated proteins were performed to immunoblotting using anti‐GST (Santa Cruz, Dallas, TX), and anti‐MBP (New England Biolabs).

### 2.12 In Vitro Ubiquitination Assay

The in vitro ubiquitination assay was conducted as described previously (Lim et al. 2018; Joo et al. 2019). Briefly, purified GST‐CaGIR1 and MBP‐CaGRAS1 proteins were mixed with 50 ng of rhUBE1 (Boston Biochemicals, Cambridge, MA, USA), 100 ng of UBCH5b (Enzo Life Sciences, Farmingdale, NY, USA), and 1 µg of bovine ubiquitin (Sigma‐Aldrich, St. Louis, MO, USA) in ubiquitination buffer solution (50 mM Tris‐HCl pH 7.5, 5 mM MgCl_2_, 2 mM dithiothreitol [DTT], and 2 mM ATP). The mixtures were incubated at 30°C for 3 h and analyzed by immunoblot analysis using anti‐GST (Santacruz), anti‐K48 linkage (Cell Signalling Technology, Danvers, MA, USA), and anti‐MBP (New England Biolabs).

### 2.13 Co‐Immunoprecipitation (Co‐IP) Assay and Protein Degradation Assay *In Planta*

To analyze the interaction between CaGRAS1 and CaGIR1, we transiently expressed *Pro35S:3xFLAG‐CaGRAS1*, *Pro35S:CaGIR1‐GFP*, and the silencing suppressor p19 in the leaves of *N. benthamiana* via agroinfiltration. As a CaGIR1 mutant lacking E3 ligase activity, CaGIR1^C59S/H61Y^ was generated by substituting cysteine 59 with serine and histidine 61 with tyrosine. Three days after infiltration, the inoculated leaves were treated with 50 μM MG132 for 12 h, after which leaf samples were collected. To analyze the protein abundance in planta, leaf total protein was extracted using native buffer (50 mM Tris‐MES pH 8.0, 0.5 M sucrose, 1 mM MgCl_2_, 10 mM EDTA pH 8.0, and 5 mM DTT) and GTEN buffer, as described previously (Baek et al. 2021). For the Co‐IP assay, the leaf total protein was incubated with anti‐FLAG magnetic beads (Sigma) and anti‐GFP magnetic beads (Chromotek, Planegg, Germany) at 4°C for 3 h. The resulting protein complexes were the subjected to immunoblot analysis using anti‐plant ubiquitin (Agrisera, Vännäs, Sweden), anti‐FLAG (Abcam), and anti‐GFP (Santacruz).

### 2.14 Cell‐Free Degradation Assay

A cell‐free degradation assay was conducted as described previously (Baek et al. 2021; Lim et al. 2022). Briefly, cell crude extracts were prepared from the leaves of *CaGIR1*‐silenced pepper plants treated with dehydration for 6 h and 100 μM ABA for 6 h using an extraction buffer (10 mM ATP, 10 mM MgCl_2_, 10 mM NaCl, 25 mM Tris‐HCl [pH 7.5], 5 mM DTT, and 0.1% Triton X‐100). Samples of the crude extract (50 μg) were incubated with GST protein and GST‐tagged CaGRAS1 for the indicated durations in the absence and presence of 50 μM MG132. After incubation, reactions were stopped by adding 4× SDS sample buffer and boiling samples for 5 min. The protein samples were separated on 10% SDS‐PAGE gels, and separation was followed by immunoblot analysis using an anti‐GST antibody.

## 3 Results

### 3.1 Drought Stress Promotes CaGRAS1 Protein Degradation via the 26S Proteasome Pathway

**Figure 1:**
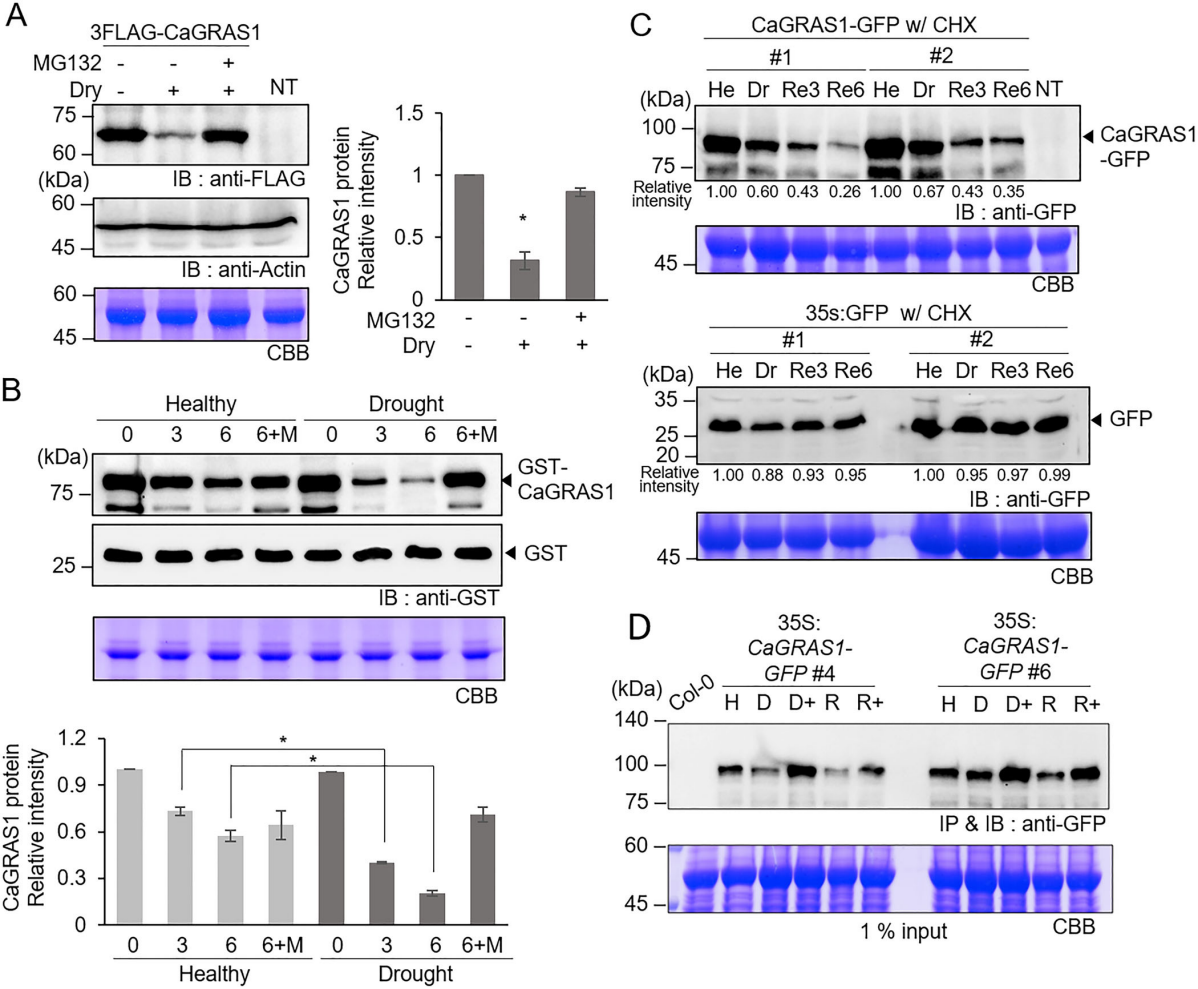
CaGRAS1 protein degraded via 26S proteasome under drought stress. (A) CaGRAS1 protein degradation under the drought stress blocked the MG132 treatment. After 2 days of inoculation of 35S:3xFLAG‐CaGRAS1, leaves harvested with 12 h of 50 μM MG132 treatment. The leaf cutting performed 6 h before sample harvesting. Anti‐actin immunoblotting and RUBISCO proteins in Coomassie brilliant blue (CBB) staining were used as loading controls. Relative intensity was calculated using the ImageJ software 1.46r (http://imagej.nih.gov/ij), with samples without MG132 and drying as a standard. (B) Cell‐free degradation assay of the CaGRAS1 protein under the 6 h drought treated leaves extract sample. The GST‐tagged CaGRAS1 protein and GST protein (negative control) were incubated with detached for 6 h 4‐week‐old pepper leaf extracts during indicated time points. Relative intensity was calculated using ImageJ software 1.46r, with each leaf extracts 0‐h sample as a standard. Data represent the mean ± SD of three independent experiments. Asterisks indicate statistically significant differences compared to the control sample (Student's *t*‐test; *p < 0.05). (C) CaGRAS1‐GFP was transiently expressed in *N. benthamiana* leaves, which were then harvested after air‐drying for 6 h (Dr) and after rewatering for 3 h (Re3) and 6 h (Re6). The samples were treated with 50 μM CHX (cycloheximide) at the start of drying to inhibit additional protein synthesis, and the relative intensity was calculated using the ImageJ programme, with the healthy (HE) sample as the standard. NT represents a non‐transgenic tobacco leaf sample. The 35S‐GFP vector control samples were incubated under the same conditions as a negative control. A CBB‐stained image was used as a loading control. Relative intensity was calculated using ImageJ software 1.46r. (D) Immunoblot analysis of CaGRAS1‐GFP overexpression lines was performed under drought (D) and recovery (R) conditions. Ten‐day‐old transgenic Arabidopsis seedlings were desiccated by placing them on 3 M paper for 30 min, followed by rewatering for 2 h by shaking in 1/2 MS medium. For MG132 treatment, seedlings were pre‐incubated in 1/2 MS medium including 50 μM MG132 for 1 h, followed by drought stress treatment (D+) and recovery with MG132 (R+). Total protein from the seedlings of each treatment was extracted using native buffer and incubated with anti‐GFP magnetic beads with equal amounts of protein, followed by immunoblotting. CBB staining showed 1% of the IP sample input, which was loaded as an internal control.

Several plant transcription factors involved in stress responses are known to undergo PTMs, such as ubiquitination, which determine their activity, abundance, and stability (Qin et al. 2008; Lee et al. 2010; Chen et al. 2013; Seo et al. 2014; Zheng et al. 2018; Singh et al. 2022). In a previous study, we found that the pepper GRAS‐type transcription factor CaGRAS1 plays a positive role in drought resistance (Oh et al. 2022). To test whether drought stress affects the stability of the CaGRAS1 protein, we transiently expressed 3xFLAG‐tagged CaGRAS1 in the leaves of *N. benthamiana* plants using agroinfiltration. After 2 days, half of the leaves were harvested as a control, whereas the other half were dried for 6 h at room temperature. In the western blot analysis, CaGRAS1 protein levels were shown to be lower in the dried leaves than in the control leaves (Figure 1A). Application of the 26S proteasome inhibitor MG132 rescued this reduction by more than 85%, which also resulted in the accumulation of ubiquitin‐conjugated CaGRAS1 proteins, as demonstrated by western blot analysis after immunoprecipitation with an anti‐FLAG antibody (Supporting Information S1: Figure S1). Additionally, we conducted a cell‐free degradation assay to analyze the effect of drought stress on CaGRAS1 protein stability. Drought stress treatments were imposed by uprooting pepper plants at the four‐leaf stage from the soil and drying them for 6 h at room temperature. GST‐tagged CaGRAS1 proteins expressed in bacterial cells were incubated with leaf crude extracts of drought‐treated and healthy pepper plants. As a result, CaGRAS1 proteins degraded more rapidly in drought‐treated samples than in the controls, and this degradation was partially inhibited by MG132 (Figure 1B). The level of GST protein, a negative control, was not different between healthy and drought samples. These results suggest that drought stress accelerates CaGRAS1 protein degradation, which occurs via the 26S proteasome pathway.

Next, we wondered how the reduction of CaGRAS1 by drought stress is modulated during recovery. To verify this, we analyzed changes in the protein levels from drought stress to recovery (water supply) using *N. benthamiana* plants transiently expressing *Pro35S:CaGRAS1‐GFP*. After 2 days of agroinfiltration, the leaf discs were collected from the infiltrated leaves treated with cycloheximide (CHX) for 6 h and then subjected to drought stress by air‐drying for 2 h, followed by rewatering for 3 or 6 h. Western blot analysis revealed a gradual decline in the CaGRAS1 protein level, with a more rapid decrease observed during the re‐watering phase (Figure 1C). As a negative control, GFP single protein showed no difference under drought stress and rewatering conditions. This reduction pattern of CaGRAS1 protein was also observed in 10‐day‐old seedlings of *Pro35S:CaGRAS1‐GFP* Arabidopsis transgenic plants (Figure 1D). CaGRAS1 protein levels declined more rapidly during the rewatering phase than during drought stress, and this degradation was partially inhibited by MG132 treatment. These findings imply that the mechanisms responsible for CaGRAS1 degradation are initiated during drought stress, and the degradation rate is significantly accelerated during recovery.

### 3.2 The Pepper RING Protein CaGIR1 Interacts With CaGRAS1

**Figure 2:**
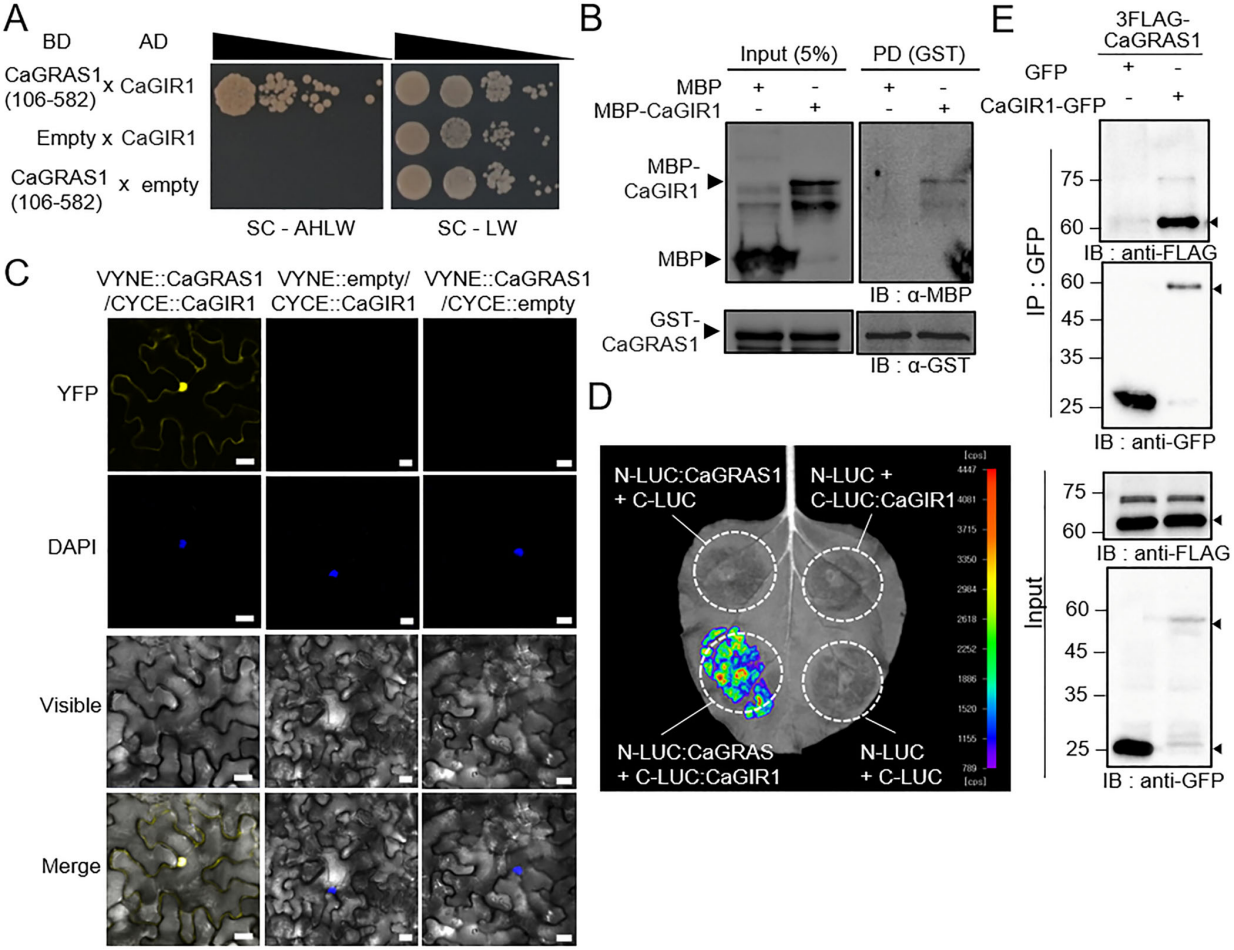
Ubiquitin E3 ligase CaGIR1 physically interacts with CaGRAS1. (A) Yeast two‐hybrid assay between truncated CaGRAS1 (aa 106‐582) and CaGIR1. To prevent auto‐activity, CaGRAS1 protein was fused with the bait vector and CaGIR1 protein was fused prey vector. Both constructs transformed as indicated combination to AH109 yeast cell that grew on the SD/‐Ade‐His‐Leu‐Trp (‐AHLW) medium and SC‐Leu/‐Trp (‐LW) medium. The interaction between the two proteins was determined by growing yeast cells on SC‐AHLW medium for 5 days. (B) CaGIR1 physically interacted with CaGRAS1 in a pull‐down assay. MBP‐CaGIR1 recombinant protein and MBP (used as a negative control) were expressed in bacterial cells, and the lysates were incubated with Sepharose resin‐bound GST‐CaGRAS1 protein. After precipitation, the samples were analyzed by western blotting using anti‐GST and anti‐MBP antibodies. Five percent of the lysate was loaded as the input sample. (C) The BiFC assay showed that CaGRAS1 interacts with CaGIR1 mainly in the nucleus. CaGRAS1 and CaGIR1 were fused with the N‐terminal and C‐terminal fragments of YFP, respectively, and transiently co‐expressed in *N. benthamiana* leaves. Empty vectors were used as a negative control. The DAPI signal (second row, blue signal) indicates the nucleus. White scale bar, 10 μm. (D) The split‐luciferase complementation assay demonstrated the interaction between CaGIR1 and CaGRAS1. The N‐terminal and C‐terminal fragments of luciferase were fused to CaGRAS1 and CaGIR1, respectively. Four pairs of combinations, including those with empty vectors, were expressed as indicated in the image. The signal was captured with a CCD camera 30 min after the injection of 0.1 M d‐luciferin. (E) Co‐immunoprecipitation analysis confirmed the interaction between CaGRAS1 and CaGIR1 in *N. benthamiana* leaf cells. Constructs of 3FLAG‐CaGRAS1, GFP, and CaGIR1‐GFP driven by the 35S promoter were co‐expressed in *N. benthamiana* leaves. Total leaf protein extracts were used as the input, and the samples precipitated with anti‐GFP magnetic beads were analyzed by western blotting using anti‐FLAG and anti‐GFP antibodies. [Color figure can be viewed at wileyonlinelibrary.com]

To isolate E3 ligases involved in CaGRAS1 protein degradation, we performed a yeast two‐hybrid assay of CaGRAS1 with the pepper RING protein small library as prey. Since the Gal4 DNA binding domain (BD) fused with full‐length CaGRAS1 has autoactivity in the yeast system (Oh et al. 2022), the N‐terminal truncated CaGRAS1 (106–582 aa) was used as bait. Of the CaGRAS1‐interacting partners, we found the putative ubiquitin E3 ligase, CA09g13790, and named it *Capsicum annuum* CaGRAS1 Interacting RING‐type E3 ligase 1 (CaGIR1) (Figure 2A). The interaction with CaGIR1 was additionally confirmed by using full‐length CaGRAS1 (Figure 2B–E). GST‐tagged CaGRAS1 and MBP‐tagged CaGIR1 were tested by pull‐down assay, which revealed that GST‐CaGRAS1 interacted with MBP‐CaGIR1, but not with MBP alone (Figure 2B). Next, to investigate the interaction between CaGRAS1 and CaGIR1 in plants, we conducted a BiFC assay and a split luciferase complementation (SLC) assay (Figure 2C,D). In the BiFC analysis, the yellow fluorescence signal for the interaction between the two proteins was shown in the cytoplasm and nucleus (Figure 2C, bottom left), whereas no signals were detected in empty vector controls (Figure 2C, bottom middle and right). The SLC assay also revealed luminescent signals only at the sites where both CaGRAS1 and CaGIR1 were expressed (Figure 2D). The interaction between CaGRAS1 and CaGIR1 was consistently shown in a Co‐IP assay (Figure 2E). In *N. benthamiana* leaves, *Pro35S:3xFLAG‐CaGRAS1* was expressed with *Pro35S:CaGIR1‐GFP* or *Pro35S:GFP* as a negative control by agroinfiltration, and we found that CaGRAS1 interacted with CaGIR1‐GFP, but not single GFP proteins, through Co‐IP and Western blot analysis. These results indicate that CaGIR1 interacts with CaGRAS1 in vivo and in vitro conditions.

### 3.3 CaGIR1 Expression Is Induced by Various Environmental Stimuli

For the molecular characterisation of CaGIR1, we initially analyzed *CaGIR1* expression in different pepper plant organs by qRT‐PCR analysis. As shown in Supporting Information S1: Figure S2A, *CaGIR1* was abundantly expressed in roots; *CaGIR1* expression levels in seedling roots were approximately 33 times higher than those of young leaves, and in mature plants at the six‐leaf stage, the levels of *CaGIR1* were 380 times higher in tap roots and 58 times higher in lateral roots than in young leaves. *CaGIR1* was also highly expressed in flowers (250 times) and fruits (30 times). We further investigated how *CaGIR1* expression is altered in response to various environmental stimuli (Supporting Information S1: Figure S2B). During the examined time period, drought stress treatment led to the gradual accumulation of *CaGIR1* transcripts, while high salinity also induced high *CaGIR1* expression after 2 h of treatment. In response to ABA and H_2_O_2_, *CaGIR1* expression levels peaked at 12 and 2 h after the treatment, respectively. These results suggest that CaGIR1 may be involved in responses to various abiotic stresses.

Since subcellular localisation is closely related to protein function, we examined which part of the cell CaGIR1 targets. In *N. benthamiana* leaves, GFP‐tagged *CaGIR1* was transiently expressed under the control of the 35S promoter through agroinfiltration. After 2 days, the GFP signals were observed in the nucleus and cytosol, in association with the sites for interaction with CaGRAS1 (Supporting Information S1: Figure S2C). The localisation of CaGIR1 was further validated in pepper protoplasts, where it was also found in both the nucleus and cytosol (Supporting Information S1: Figure S3).

### 3.4 CaGIR1 Plays a Role as a Ubiquitin E3 Ligase

**Figure 3:**
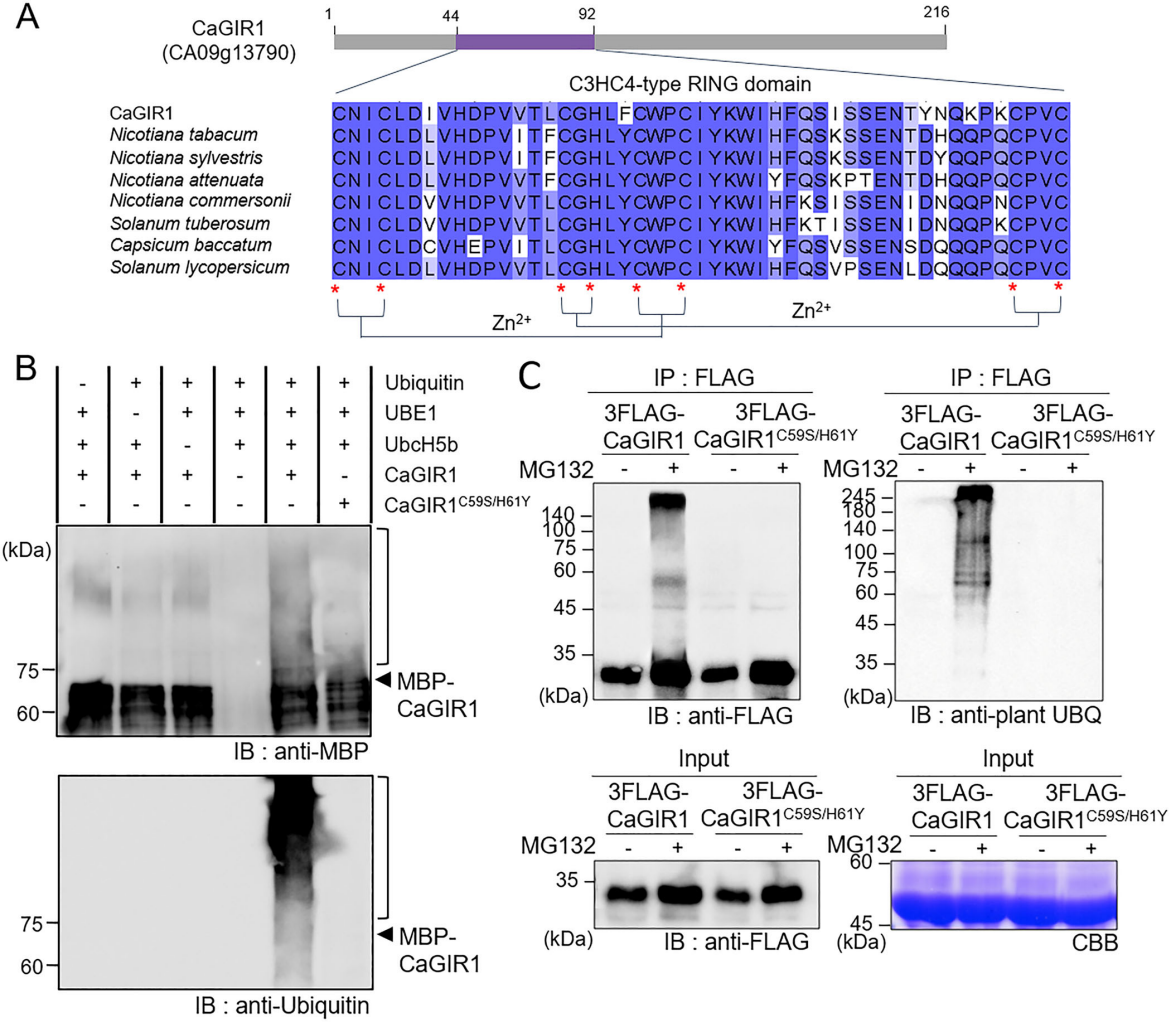
CaGIR1 is a functional ubiquitin E3 ligase. (A) C3HC4‐type RING domain of the CaGIR1 protein. Multiple sequence alignment analysis was performed with amino acid residues of the RING domains of CaGIR1 and its homologous proteins from other plants belonging to the *Solanaceae* family. Amino acids were shaded based on percentage identity in ClustalW2. The red asterisks indicate cysteine and histidine residues that bind to zinc ions, thus forming zinc finger structures. (B) In vitro self‐ubiquitination assay of CaGIR1. Western blot analysis was conducted to detect ubiquitinated MBP‐CaGIR1 using anti‐MBP and anti‐ubiquitin antibodies. The CaGIR1^C59S/H61Y^ protein was used as a RING domain‐defective mutant. (C) In vivo ubiquitination assay of CaGIR1 and CaGIR1^C59S/H61Y^ to asses E3 ligase activity mediated by the RING domain. Leaf extracts from *N. benthamiana* plants expressing *3FLAG‐CaGIR1* and CaGIR1^C59S/H61Y^ were used for immunoprecipitation with anti‐FLAG magnetic beads. A solution of 50 μM MG132 was infiltrated 12 h before leaf sampling to block the degradation of ubiquitinated proteins. Total leaf protein extracts were used as the input, and the samples precipitated with anti‐FLAG magnetic beads were analyzed by western blot analysis using anti‐FLAG and anti‐plant ubiquitin antibodies. Coomassie brilliant blue (CBB) staining indicates that all protein samples were equally loaded. [Color figure can be viewed at wileyonlinelibrary.com]

According to the domain analysis conducted using SMART (Simple Modular Architecture Research Tool; http://smart.embl‐heidellberg.de), CaGIR1 has a C3HC4‐type RING zinc finger domain spanning amino acid 44–92 that shares high sequence homology with the RING domains of other plants belonging to the *Solanaceae* family (Figure 3A). Accordingly, we expected CaGIR1 to be a functional ubiquitin E3 ligase. An in vitro ubiquitination assay was conducted to confirm whether the CaGIR1 protein has E3 ligase activity. As shown in Figure 3B, MBP‐CaGIR1 fusion proteins were mixed in various combinations with ubiquitination components, such as E1 (UBE1, recombinant) and E2 (UbcH5b, recombinant), and bovine ubiquitin. As a result of Western blot analysis using anti‐MBP and anti‐ubiquitin antibodies, the high molecular weight smear was observed on Lane 5, which contained CaGIR1 proteins and all ubiquitination components, indicating that CaGIR1 proteins were polyubiquitinated. In contrast, no ubiquitination signals were detected in the CaGIR1 RING domain mutant (CaGIR1^C59S/H61Y^) generated by substituting cysteine 59 and histidine 61 for serine and tyrosine, respectively. The same pattern was observed when any of the components were missing. We further investigated in vivo ubiquitination of CaGIR1 proteins by expressing CaGIR1‐GFP and CaGIR1^C59S/H61Y^‐GFP in *N. benthamiana* leaves (Figure 3C,D). After 2 days of agroinfiltration, the application of MG132 resulted in an increase in CaGIR1 protein abundance as well as the accumulation of ubiquitinated CaGIR1. Consistently, the high molecular weight smear observed above the CaGIR1 protein band was not caused by expression of the CaGIR1^C59S/H61Y^ mutant protein (Figure 3D). These results suggest that CaGIR1 functions as an active E3 ubiquitin ligase and that this function is dependent on its RING domain.

### 3.5 CaGIR1 Directly Ubiquitinates CaGRAS1 as a Substrate

**Figure 4:**
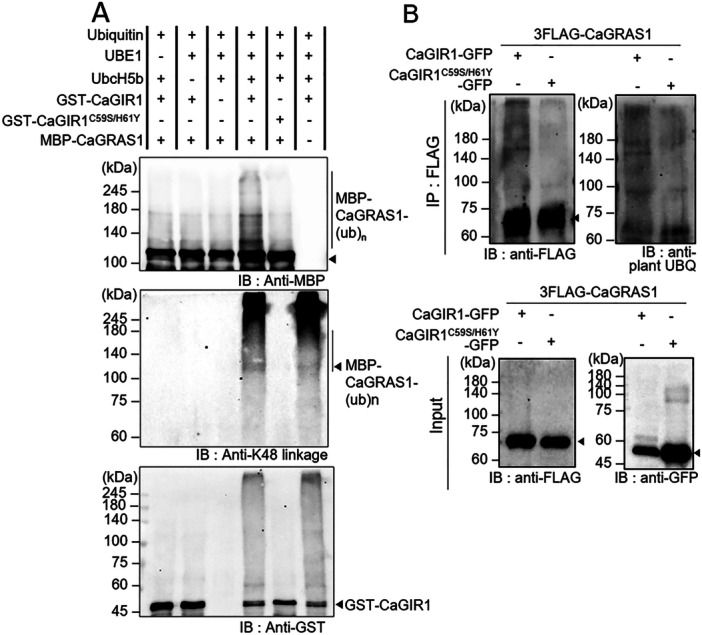
CaGIR1‐mediated ubiquitination of CaGRAS1 in vitro and in vivo. (A) In vitro ubiquitination assay of CaGRAS1 by CaGIR1. MBP‐CaGRAS1 was incubated with GST‐CaGIR1 in the presence of UBE1 (E1), UbcH5b (E2), and bovine ubiquitin. Immunoblot analyses were performed using anti‐K48 linkage (top), anti‐MBP (middle), and anti‐GST (bottom) antibodies. (B) In vivo ubiquitination assay of CaGRAS1 by CaGIR1. In *N. benthamiana* leaves, 3xFLAG‐CaGRAS1 was co‐expressed with CaGIR1‐GFP or CaGIR1^C59S/H61Y^‐GFP. Leaf protein samples were precipitated with anti‐FLAG magnetic beads and analyzed by Western blotting with the indicated antibodies. The input figure indicates the target protein levels in total protein extracts, loaded in equal amounts.

Since CaGIR1 has E3 ubiquitin ligase activity and interacts with CaGRAS1, we hypothesised that CaGRAS1 could be a substrate of CaGIR1. To investigate whether CaGIR1 ubiquitinates CaGRAS1 proteins, we conducted an in vitro ubiquitination assay by adding GST‐CaGIR1 as an E3 ligase and MBP‐CaGRAS1 as a substrate. As shown in Figure 4A, a faint smear of higher molecular weight appeared above CaGRAS1 (Lane 4) on the anti‐MBP immunoblots, but no such smear was present in the absence of GST‐CaGIR1 (Lane 3) or ubiquitination enzymes (UBE1, and UbcH5b. Lanes 1–2). The anti‐K48 linkage immunoblot signals indicated a significant ubiquitin‐ladder signal, suggesting an active proteolytic reaction. This CaGIR1‐mediated ubiquitination of CaGRAS1 proteins also occurred in plants (Figure 4B). CaGIR1‐GFP and 3xFLAG‐CaGRAS1 were co‐expressed in *N. benthamiana* leaves and, following immunoblot analysis with anti‐FLAG and anti‐plant ubiquitin antibodies, the higher molecular weight smear was observed above 70 kDa. As expected, the expression of the CaGIR1^C59SH61Y^‐GFP mutant did not induce polyubiquitination of CaGRAS1. These data indicate that CaGIR1 is an active E3 ubiquitin ligase that directly ubiquitinates CaGRAS1.

### 3.6 Silencing of *CaGIR1* Enhances Drought Resistance in Pepper Plants

**Figure 5:**
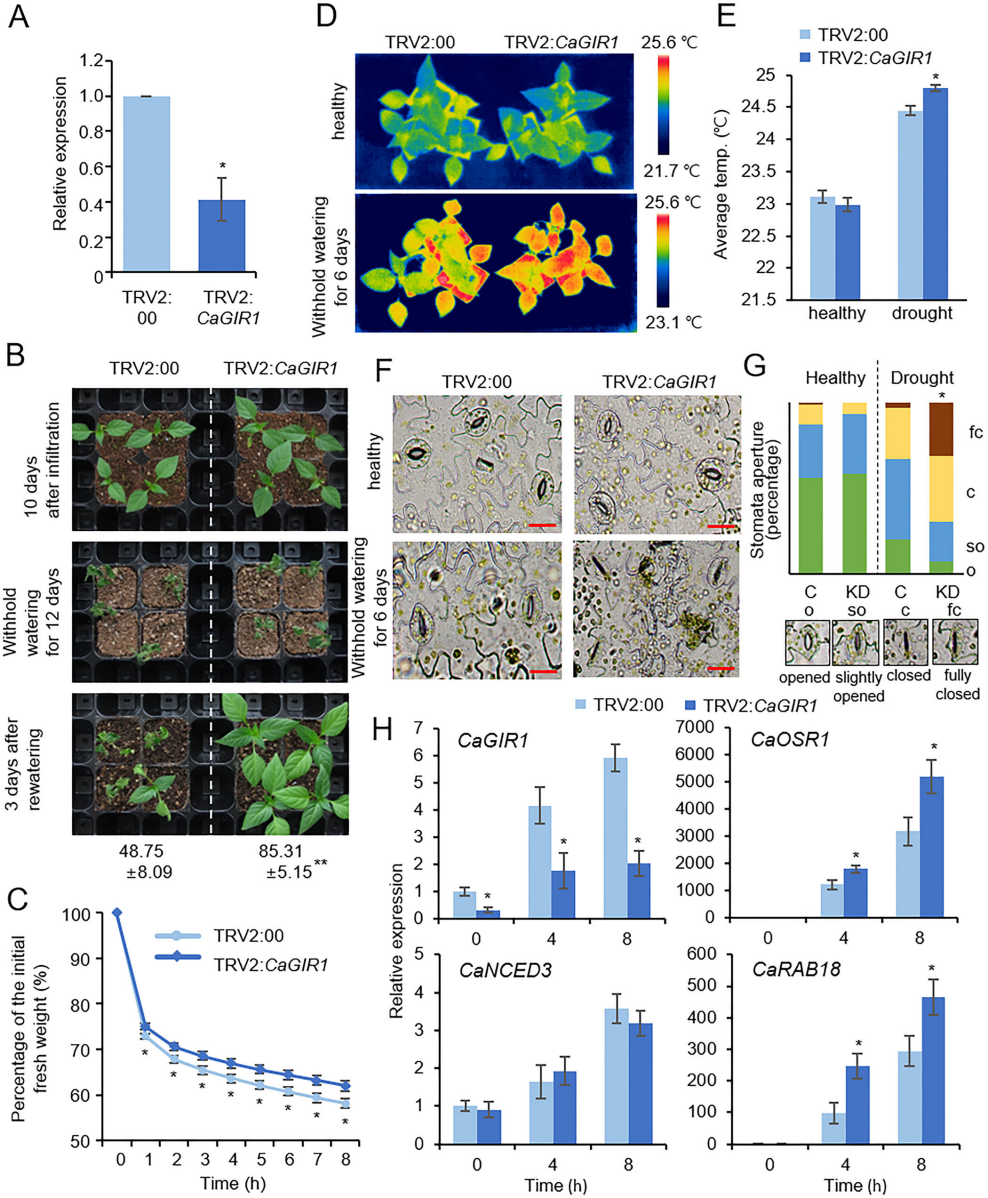
Enhanced resistance of *CaGIR1*‐silenced pepper plants to drought stress. (A) Expression level of *CaGIR1* in the leaves of *CaGIR1*‐silenced pepper plants (TRV2:*CaGIR1*) and control plants (TRV2:00). *CaACT1* was used as an internal control for normalisation. (B) Enhanced resistance of *CaGIR1*‐silenced pepper plants to drought stress. Ten days after agroinfiltration, TRV2:*CaGIR1* and TRV2:00 pepper plants grown under well‐watered conditions (upper) were subjected to drought stress by withholding water for 12 days (middle). After rewatering for 3 days (bottom), we determined the survival rates of each plant line. (C) Transpirational water loss from the detached leaves of TRV2:*CaGIR1* and TRV2:00 pepper plants. After leaf detachment, leaf fresh weights of each plant line (*n* ≥ 30 plants per line) were measured at the indicated time points. (D and E) Leaf temperature of TRV2:00 and TRV2:*CaGIR1* pepper plants after withholding the water for 6 days as drought stress. Control and CaGIR1‐silenced pepper plants were subjected to drought stress by withholding watering for 6 days, then representative thermographic images were taken (D), and leaf temperature was measured (E). (F and G) drought‐affected stomatal closure in control and CaGIR1‐silenced pepper plants. Two‐week‐old control and TRV2:CaGIR1 plants were subjected to withhold water for 6 days. Representative images were taken (F) and the stomatal apertures (*n* > 100) and measured which was indicated (G). The scale bar represents 20 μm. (H) Expression patterns of drought‐responsive marker genes in the leaves of TRV2:00 and TRV2:*CaGIR1* pepper plants in response to drought stress. The expression levels of each gene in TRV2:00 plants at 0 h were set to 1.0. The relative expression levels (ΔΔCT) of each gene were normalised to that of the *CaACT1* gene, which served as an internal control. All data represent the mean ± standard error of three independent experiments. Asterisks indicate significant differences between TRV2:00 and TRV2:*CaGIR1* pepper plants (Student's *t*‐test; *p < 0.05). [Color figure can be viewed at wileyonlinelibrary.com]

We explored the biological function of CaGIR1 in response to drought stress as an interacting partner of CaGRAS1. Because VIGS methods are effective for the functional analysis of pepper genes, we applied this method to generate *CaGIR1*‐silenced pepper plants (Figure 5). At 2 weeks after agroinfiltration, qRT‐PCR analysis revealed that the *CaGIR1* transcript level was significantly lower in the leaves of TRV2:*CaGIR1* than in those of TRV2:00 control pepper plants (Figure 5A). Next, TRV2:*CaGIR1* and TRV2:00 plants were subjected to drought stress by withholding watering for 12 days. As a result of rewatering for 3 days, more TRV:*CaGIR1* pepper plants were rescued and reestablished than TRV2:00 plants, and, consistently, the survival rate of TRV:*CaGIR1* was approximately 1.8 times higher than that of TRV2:00 plants (Figure 5B). To determine whether the silencing of *CaGIR1* affects transpiration regulation, we measured the fresh weights of leaves from the two plant lines during the indicated time periods after leaf detachment. As shown in Figure 5C, transpirational water loss was lower in TRV2:*CaGIR1* than in TRV2:00 plants. Transpiration regulation is tightly associated with stomatal opening and closing. Because leaf surface temperatures are indirect indicators of these stomatal responses to environmental changes, we measured the leaf surface temperatures of TRV2:*CaGIR1* and TRV2:00 plants after treatment with soil‐drying for 6 days (Figure 5D,E). The leaf surface temperature of TRV2:*CaGIR1* and TRV2:00 plants did not differ under normal conditions, but drought stress elevated the leaf temperature of TRV2:*CaGIR1* plants above that of TRV2:00 plants. A stomatal aperture assay also demonstrated similar results (Figure 5F,G); under normal conditions, the stomatal apertures of the two plant lines did not differ, and their stomatal apertures decreased with drought stress, but the sizes of stomatal apertures in *TRV2:CaGIR1* was smaller than those in TRV2:00 plants when withholding water for 6 days. Moreover, we examined whether the silencing of the *CaGIR1* gene affected drought‐responsive marker gene expression (Figure 5H). In response to drought stress, *CaGIR1* gene expression increased, but its expression level was significantly lower in TRV2:*CaGIR1* than in TRV2:00 at all examined time points. The expression of three marker genes increased after drought stress treatment, and *CaOSR1*, a *RD29B* homologue gene in pepper, and *CaRAB18* were expressed at a higher level in TRV2:*CaGIR1* than in TRV2:00, with the exception of *CaNCED3*. These results indicate that CaGIR1 may contribute negatively to drought resistance by altering ABA‐mediated stomatal responses along with drought‐responsive gene expression.

### 3.7 Overexpression of *CaGIR1* Reduces ABA Sensitivity in Arabidopsis Plants

To further investigate the biological functions of CaGIR1, we generated Arabidopsis transgenic plants overexpressing *CaGIR1* under the control of the 35S promoter. Two independent lines (#3 and #5) of *Pro35S:CaGIR1* were selected, and overexpression of *CaGIR1* in their seedlings was confirmed by qRT‐PCR analysis (Supporting Information S1: Figure S4A). Using these lines, we initially analyzed how CaGIR1 plays a role in ABA‐mediated regulation of seed germination and seedling growth. In the absence of ABA, *Pro35S:CaGIR1* seeds germinated at a similar level to wild‐type seeds (Supporting Information S1: Figure S4B). In contrast, *Pro35S:CaGIR1* seeds showed higher germination rates than wild‐type seeds when placed on 1/2 MS media containing 0.5 and 0.75 μM ABA. This reduced ABA sensitivity was also observed in seedling establishment and root growth at 7 days after plating; compared to wild‐type seedlings, *Pro35S:CaGIR1* seedlings showed higher cotyledon greening (Supporting Information S1: Figure S4C,D) and root length (Supporting Information S1: Figure S4E,F). We also tested whether overexpression of the *CaGIR1* gene affects seedling growth after germination (Supporting Information S1: Figure S4G,H). There was no difference in growth between wild‐type and *Pro35S:CaGIR1* seedlings in the absence of ABA. In contrast, the roots of *Pro35S:CaGIR1* seedlings were longer than those of wild‐type plants 5 days after 2‐day‐old seedlings had been transplanted into 1/2 MS media containing 20 μM ABA. These data suggest that CaGIR1 may play a negative role in ABA‐mediated inhibition of seed germination and post‐germination growth.

### 3.8 Overexpression of *CaGIR1* Reduces Drought Resistance in Arabidopsis

**Figure 6:**
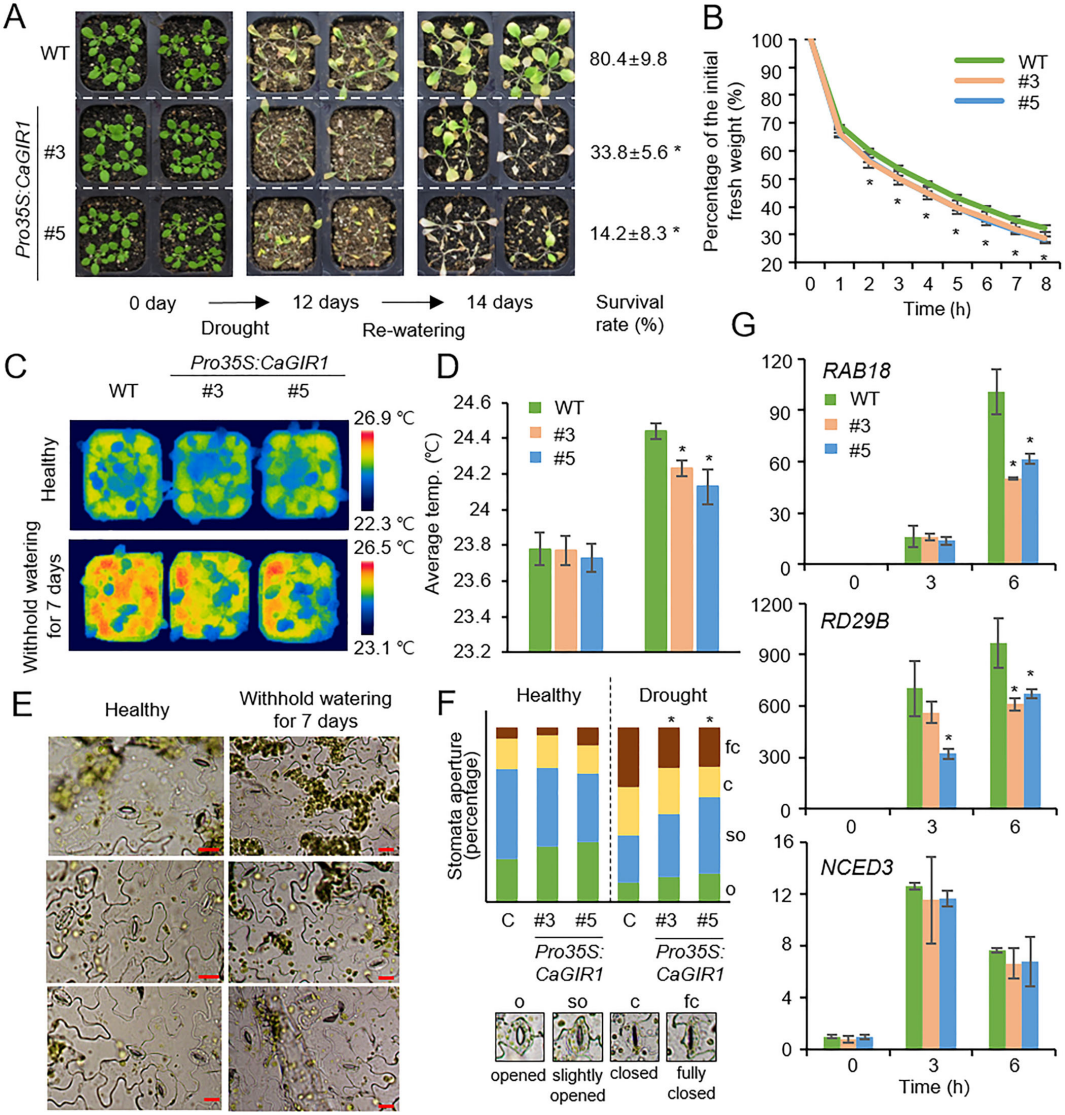
Reduced resistance of *CaGIR1*‐overexpressing Arabidopsis plants to drought stress. (A) Reduced resistance of *Pro35S:CaGIR1* Arabidopsis plants to drought stress. Three‐week‐old *Pro35S:CaGIR1* and wild‐type (WT) plants grown under well‐watered conditions (left) were subjected to drought stress by withholding water for 12 days (middle). After rewatering for 2 days (right), we determined the survival rates of each plant line. (B) Transpirational water loss from the detached leaves of *Pro35S:CaGIR1* and WT plants. After leaf detachment, leaf fresh weights of each plant line (*n* ≥ 30 plants per line) were measured at the indicated time points. (C and D) Representative thermographic images were taken for the leaf temperatures of wild‐type and *Pro35S:CaGIR1* transgenic plants after withholding water supply for 7 days. (E and F) Stomatal apertures measurement in wild‐type and *Pro35S:CaGIR1* plants from healthy and withholding for 7 days. Representative images were taken under a microscope (E) and the stomatal apertures were measured (F). The scale bar represents 20 μm. (G) Expression patterns of drought‐responsive marker genes in the leaves of *Pro35S:CaGIR1* and WT plants in response to drought stress. The expression levels of each gene in WT plants at 0 h were set to 1.0. The relative expression levels (ΔΔCT) of each gene were normalised to that of the *AtACT8* gene, which served as an internal control. All data represent the mean ± standard of three independent experiments. Asterisks indicate significant differences between *Pro35S:CaGIR1* and WT plants (Student's *t*‐test; *p < 0.05). [Color figure can be viewed at wileyonlinelibrary.com]

Next, we assessed the drought stress responses of *Pro35S:CaGIR1* plants (Figure 6). Three‐week‐old seedlings of *Pro35S:CaGIR1* and wild‐type plants were subjected to drought stress by withholding water for 12 days (Figure 6A). Under well‐watered conditions, both plants exhibited no significant difference in growth (Figure 6A, left panel). However, *Pro35S:CaGIR1* plants were more wilted after drought stress treatment than wild‐type plants (Figure 6A, middle panel). After 2 days of rewatering, 14.2%–33.8% of *Pro35S:CaGIR1* plants survived and resumed their growth, whereas 80.4% of wild‐type plants recovered (Figure 6A, right panel). For *Pro35S:CaGIR1* leaves, fresh weights were lower after detachment than wild‐type leaves, meaning that *CaGIR1* overexpression enhanced transpirational water loss (Figure 6B). Consistently, drought stress resulted in lower leaf temperatures (Figure 6C,D) and larger stomatal apertures (Figure 6E,F) in *Pro35S:CaGIR1* leaves than in wild‐type leaves. Under the well‐watered condition, there was no difference in leaf temperatures and stomatal apertures between plant lines. Drought‐responsive marker gene expression was also found to contribute to reduced drought resistance. *RAB18* and *RD29B* gene expression was strongly induced by drought stress in both *Pro35S:CaGIR1* and wild‐type plants, but their expression levels were significantly lower in *Pro35S:CaGIR1* plants (Figure 6G). In contrast, there was no difference in *NCED3* expression between plant lines. These data indicate that overexpression of *CaGIR1* reduces drought resistance by modulating ABA‐mediated stomatal responses and drought‐responsive marker gene expression.

### 3.9 CaGIR1 Modulates CaGRAS1 Protein Stability in Response to Drought Stress and ABA

**Figure 7:**
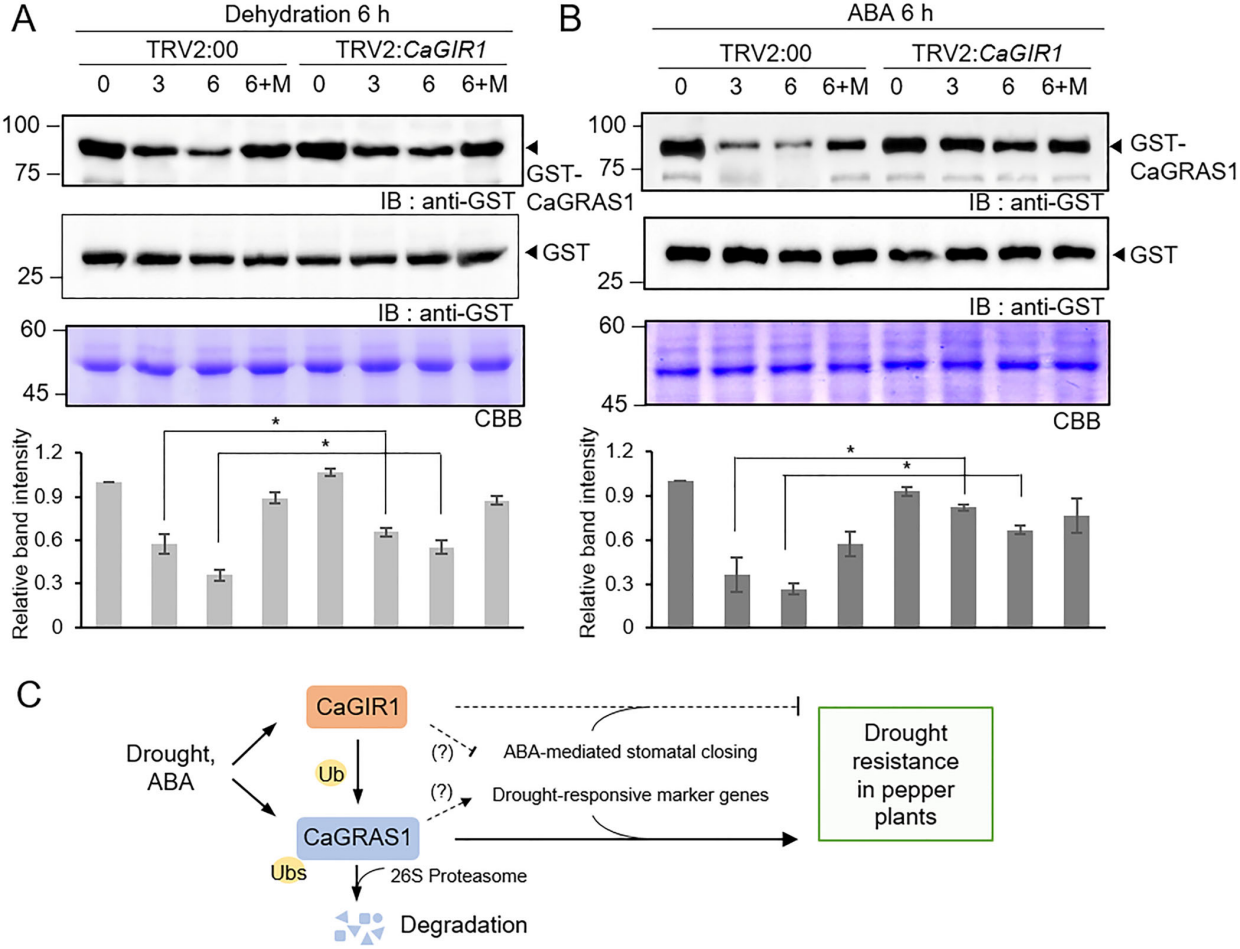
Regulation of CaGRAS1 protein stability by CaGIR1. (A and B) Cell‐free degradation assay of CaGRAS1 proteins. GST and GST‐tagged CaGRAS1 fusion proteins were incubated with the leaf crude extracts of TRV2:*CaGIR1* and TRV2:00 plants treated with drought stress for 6 h (A) and 100 μM ABA for 6 h (B). MG132 was used as an inhibitor of the 26S proteasome. The GST protein was used as a negative control. Coomassie brilliant blue (CBB) staining indicates that all protein samples were equally loaded. Relative band intensity was calculated with ImageJ software 1.46r, and data represent the mean ± standard of three independent experiments. Asterisks indicate significant differences between TRV2:*CaGIR1* and TRV2:00 pepper plants (Student's *t*‐test; *p < 0.05). (C) A representative schematic on functional role of CaGIR1 and CaGRAS1 in response to drought stress and ABA. A schematic representation illustrating the functional roles of CaGIR1 and CaGRAS1 in response to drought stress and ABA. An arrow represents enhancement and the end bar indicates suppression. [Color figure can be viewed at wileyonlinelibrary.com]

In response to drought stress, CaGRAS1 protein degradation was triggered (Figure 1B) and *CaGIR1* expression was highly induced (Supporting Information S1: Figure S2B). Based on these results, we wondered whether CaGIR1 participates in the degradation of CaGRAS1 proteins under drought stress. To explore this possibility, we performed a cell‐free degradation assay using leaf crude extracts from *CaGIR1*‐silenced pepper plants (Figure 7). TRV2:*CaGIR1* and TRV2:00 plants were subjected to drought stress for 6 h as described above, and leaf samples were harvested for isolation of crude extracts. We expressed GST‐CaGRAS1 fusion proteins in bacterial cells and then incubated samples for the indicated times with leaf crude extracts of each plant line. As shown in Figure 7A, GST‐CaGRAS1 protein degradation occurred less in crude extracts from TRV2:*CaGIR1* plants compared to those from TRV2:00 plants. Following incubation, CaGRAS1 protein levels were approximately 2–3 times higher in TRV2:*CaGIR1* than in TRV2:00. This protein degradation was restored partially by the application of MG132. Since drought stress induces an increase in ABA content in plant leaves, we sprayed 100 μM ABA on TRV2:*CaGIR1* and TRV2:00 leaves and isolated the leaf crude extracts for a cell‐free degradation assay (Figure 7B). Similarly, the levels of CaGRAS1 proteins in TRV2:*CaGIR1* were approximately 1.5 and 2 times higher than in TRV2:00 after incubation for 3 and 6 h, respectively. Additionally, MG132 treatment partially inhibited CaGRAS1 protein degradation, but this effect was not clearly evident in TRV2:*CaGIR1* samples. These results suggest that CaGRAS1 protein stability is modulated by CaGIR1 in ABA‐mediated drought stress responses.

## 4 Discussion

In previous study, we identified the GRAS‐type transcription factor CaGRAS1, which plays a positive role in ABA signalling and drought responses (Oh et al. 2022). As an interacting partner of CaGRAS1, the C3HC4 RING‐type E3 ligase CaGIR1 was isolated in a yeast two‐hybrid assay with a pepper RING protein small library, and the interaction between the two proteins was confirmed by in vivo and in vitro experiments (Figure 2). As expected, CaGIR1 was found to have E3 ligase activity that was dependent on its RING domain, leading to the ubiquitination of CaGRAS1 proteins (Figure 7C). Though CaGRAS1 is a member of the PHYTOCHROME A SIGNAL TRANSDUCTION1 (PAT1) subfamily, research on GRAS protein regulation by ubiquitination has mostly focused on the DELLA subfamily proteins, which acts as a repressor of the GA receptor in the GA signalling pathway (Xu et al. 2015; Yuan et al. 2016; Zhang et al. 2020; Hernández‐García et al. 2021). The DELLA‐GID1(GA)‐SLY1 complex initiates GA signalling by triggering 26S proteasome‐mediated DELLA protein degradation. In this event, the F‐box protein SLY1 recognises the GRAS domain on DELLA protein, GID1, which makes it possible to form a complex and subsequently degrade its target protein (McGinnis et al. 2003; Dill et al. 2004). The FKF1 F‐box protein also degrades the DELLA protein GAI and RGA proteins, enabling the regulation of flowering time under long‐day conditions (Yan et al. 2020). Moreover, the COP1 E3 ligase complex is responsible for the degradation of the DELLA protein RGA in Arabidopsis (Blanco‐Tourinan et al. 2020; Lee et al. 2022). The protein stability of OsSCL7, a member of the SCARECROW subfamily of GRAS proteins, is subject to regulation through the 14‐3‐3 protein GF14c, and this process impacts plant immunity (Lu et al. 2022). These findings suggest that the stability of various GRAS subfamily proteins is regulated by ubiquitination in response to biotic and abiotic stresses.

Through phenotypic analysis using *CaGIR1*‐silenced pepper plants and *CaGIR1*‐overexpressing transgenic Arabidopsis plants, we demonstrated that CaGIR1 acts as a negative regulator in response to drought stress. These phenotypic changes caused by CaGIR1 are the opposite of the changes caused by its interacting partner CaGRAS1 (Oh et al. 2022). Compared to the control pepper plants, *CaGIR1*‐silenced pepper plants displayed enhanced drought resistance through modulated stomatal responses and drought‐responsive marker gene expression (Figure 5), while *CaGRAS1*‐silenced pepper plants were highly sensitive to drought stress, as characterised by ABA insensitivity. Consistently, *CaGIR1*‐overexpressing transgenic Arabidopsis plants showed reduced drought resistance as well as an ABA hyposensitive phenotype during seed germination and seedling growth compared to wild‐type plants (Figure 6 and Supporting Information S1: Figure S4). In contrast, *CaGIR1*‐overexpressing showed drought‐resistant and ABA‐sensitive phenotypes. Based on these findings, CaGRAS1 may be regulated by CaGIR1 in response to ABA and drought stress.

Although CaGRAS1 acts as a positive regulator of drought resistance in pepper plants, drought stress appears to promote the degradation of CaGRAS1 proteins. In a cell‐free degradation assay, CaGRAS1 proteins were rapidly degraded when incubated with leaf crude extracts from pepper plants exposed to drought stress compared to when they were incubated with crude extracts from pepper plants not exposed to drought stress (Figure 1). This CaGRAS1 protein degradation is significantly accelerated even under rewatering conditions. Several studies have reported that stress‐related genes are regulated by E3 ligases, many of which act as negative regulators of stress responses. For instance, the RING E3 ligase RGLG2 in Arabidopsis interacts with ERF53, thereby suppressing the plant's drought stress response (Cheng et al. 2012). In addition, DRIP1 and DRIP2 function as negative regulators of drought‐responsive gene expression by directing DREB2A for 26S proteasome‐mediated degradation (Qin et al. 2008). These findings suggest that E3 ligases fine‐tune stress responses by degrading drought stress‐related proteins, thereby ensuring proper stress regulation and facilitating the transition back to normal growth. The potential involvement of CaGIR1 in CaGRAS1 protein degradation was demonstrated by two points. First, CaGRAS1 protein stability was affected by *CaGIR1* expression levels. A cell‐free degradation assay revealed that the degradation of CaGRAS1 proteins was suppressed upon incubation with leaf crude extracts from *CaGIR1*‐silenced pepper plants exposed to drought stress, as compared to incubation with control plant extracts (Figure 7A). A similar pattern was observed using leaf crude extracts from *CaGIR1*‐silenced pepper plants treated with ABA (Figure 7B). Combined with the results of in vitro and in vivo ubiquitination assays (Figure 4), these results suggest that CaGIR1‐mediated ubiquitination regulates CaGRAS1 protein stability in response to ABA and drought stress. Second, the involvement of CaGIR1 in drought stress responses and CaGRAS1 degradation is further supported by *CaGRAS1* and *CaGIR1* genes exhibiting different expression patterns in response to ABA and drought stress. In particular, *CaGRAS1* expression appeared to be induced earlier than *CaGIR1* expression. In response to drought stress and ABA treatment, *CaGRAS1* gene expression was rapidly induced, reaching its maximum expression level within 2–6 h, whereas *CaGIR1* exhibited a delayed response, with its expression level gradually increasing over 24 h and peaking at 12 h. Based on these results, we propose that CaGIR1 functions to promote the degradation of CaGRAS1, a stress‐related transcription factor, thus facilitating the resumption of normal growth.

In conclusion, we demonstrated that CaGIR1 plays a role as a negative regulator in drought resistance via modulating the protein stability of the CaGRAS1 transcription factor. Although CaGIR1 promotes the degradation of CaGRAS1 during drought stress, when the plants are rewatered, this process may facilitate the transition back to normal growth and development. This suggests that CaGIR1 helps the plant switch from a stress response mode to a recovery phase by regulating CaGRAS1 levels. However, further studies are needed to fully understand the mechanisms underlying the regulation of CaGRAS1 protein stability, as the degradation of CaGRAS1 by CaGIR1 appears to be partial. Given the stability of the ABI5 protein, which is regulated by multiple E3 ligases, it is possible that additional pepper E3 ligases apart from CaGIR1 may also play a role in regulating CaGRAS1 protein stability in response to ABA and drought stress. The identification of E3 ligases and PTMs of CaGRAS1 proteins will offer novel insights into the mechanisms of plant survival mediated by CaGRAS1 against environmental stress.

## Conflicts of Interest

The authors declare no conflicts of interest.

## Supporting information

Supplementary Data.

## Data Availability

The data that support the findings of this study are available in the Supporting Information of this article.
