# Supplementary material for: The Pepper E3 Ligase CaGIR1 Acts as a Negative Regulator of Drought Response via Controlling CaGRAS1 Stability
Source: Plant Cell Environ. 2025 Apr 8;48(7):5498–513. doi: 10.1111/pce.15516 (PMC12131955; doi:10.1111/pce.15516)
Supplement: Supplementary file 1 — Supplementary Data. [file PCE-48-5498-s001.pdf]

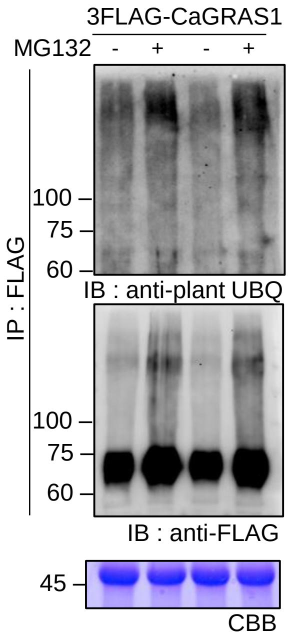

Supplementary figure 1. Transiently expressed 3xFLAG-CaGRAS1 protein showed the dragged band in after 12h 50 μM MG132 treatment sample. After 2 days of infiltration, inoculated leaves harvested with 12 hours of 50 μM MG132 treatment. Immunoblot analysis and immunoprecipitation analysis were subjected using Anti-FLAG, and Anti-plant ubiquitin antibody. RUBISCO proteins in Commassie blue staining were used as a loading control.

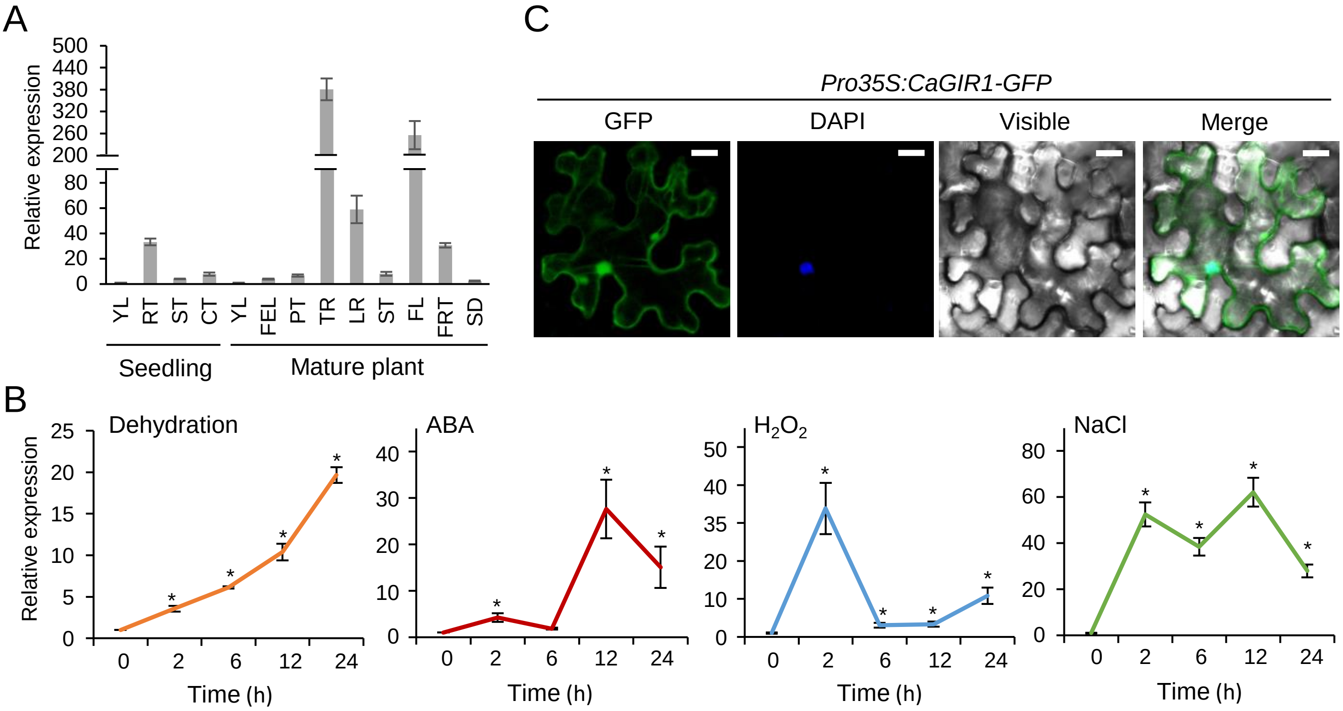

Supplementary figure 2. Molecular characterization of CaGIR1. (a) Organ-specific expression of *CaGIR1* in 3-week-old pepper seedlings and mature plants at the six-leaf stage. The expression level of *CaGIR1* in YL was set to 1.0. YL, young leaf; RT, root; ST, stem; CT, cotyledon; FEL, fully expanded leaf; PT, petiole; TR, tap root; LR, lateral root; FL, flower; FRT, fruit; SD, seed. (b) Expression patterns of *CaGIR1* in the leaves of pepper plants after treatment with drought, NaCl (200 mM), ABA (100 μM), or H_2O_2 (100 μM). The pepper *Actin1* (*CaACT1*) gene was used as an internal control. Asterisks indicate significant differences compared to the untreated sample (0 h) (Student's *t*-test; **P* < 0.05). (c) Subcellular localization of CaGIR1 proteins. A *Pro35S:CaGIR1-GFP* fusion construct was expressed in *N. benthamiana* leaves using agroinfiltration, and after 2 days, the green fluorescent protein (GFP) signal was observed under a confocal laser scanning microscope. DAPI staining was used as a marker for the nucleus. White bar, 10 μm.

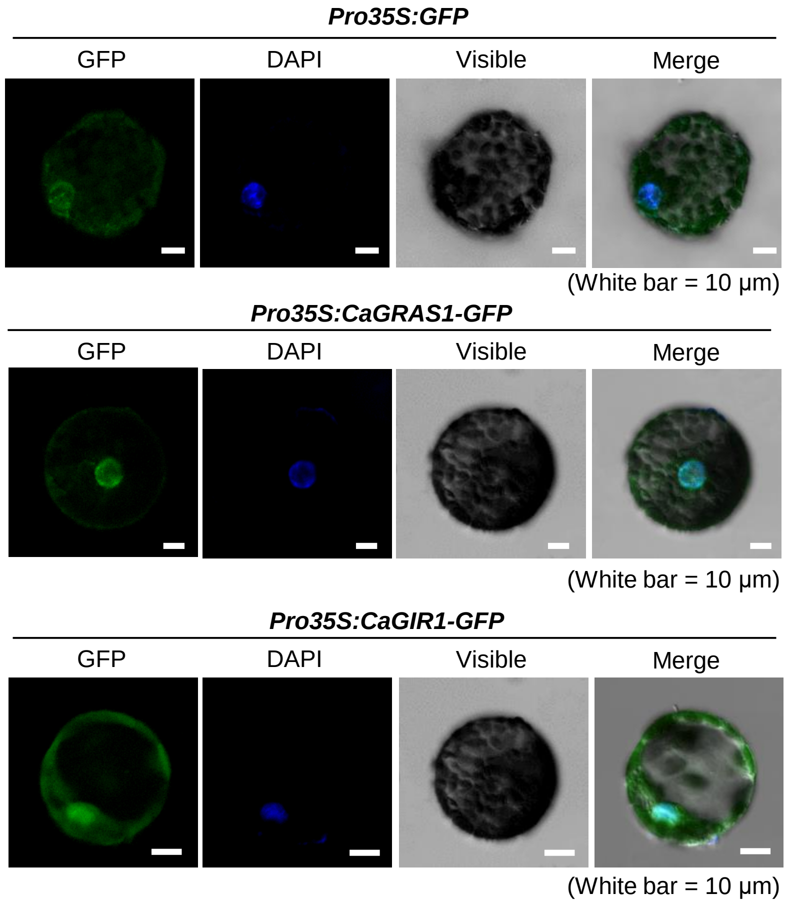

Supplementary figure 3. Cellular localization of CaGIR1 protein in pepper protoplasts. Each construct was introduced into isolated pepper protoplasts via DNA transfection and subsequently observed using a confocal microscope (Carl Zeiss LM700). Expression of *Pro35S:GFP* (top, as a negative control), *Pro35S:CaGRAS1-GFP* (middle) and *Pro35S:CaGIR1-GFP* (bottom). Scale bars, 10 µm.

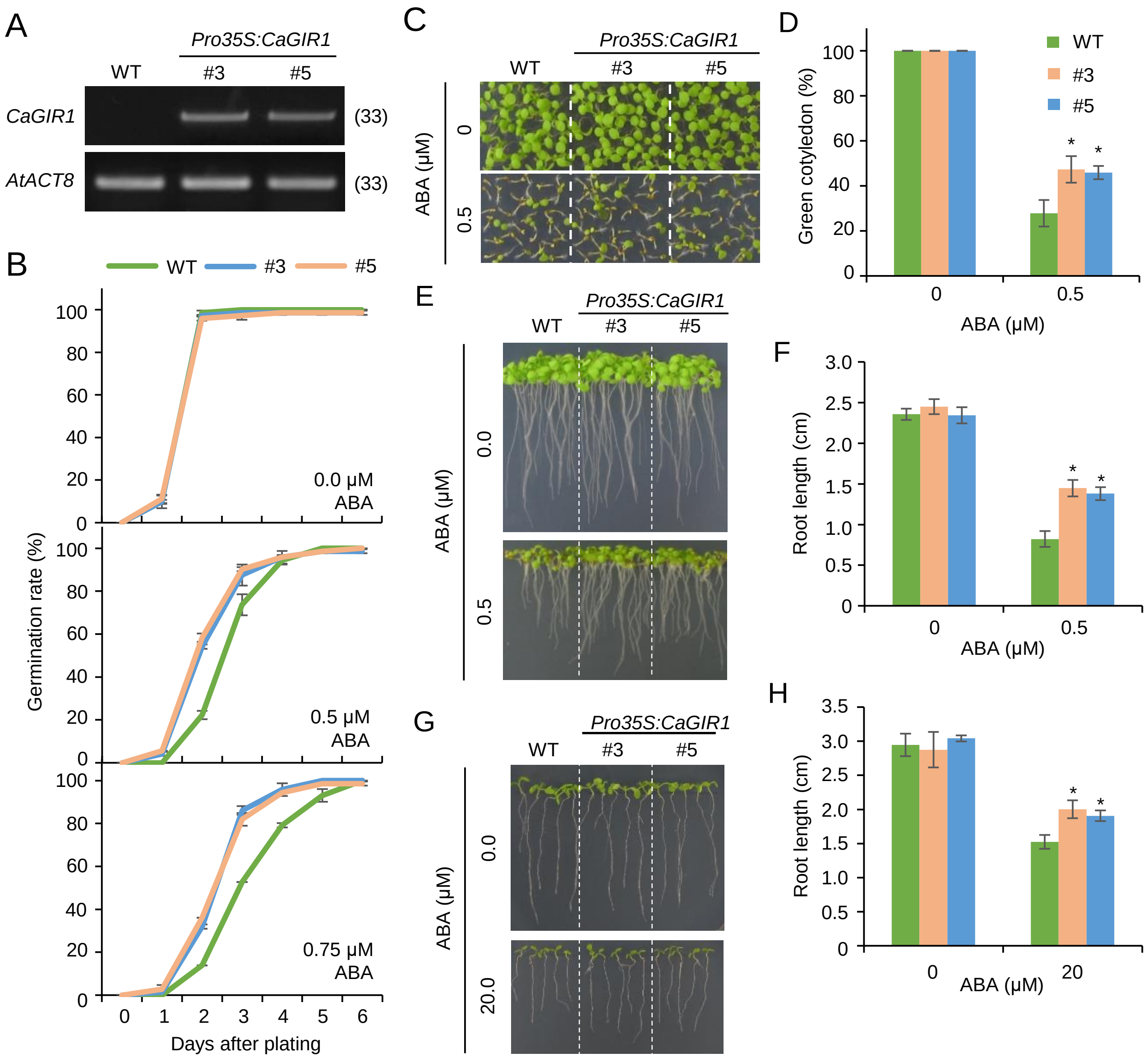

Supplementary figure 4. Reduced ABA sensitivity of *CaGIR1*-overexpressing Arabidopsis transgenic plants during seed germination and post-germination stages. (a) RT-PCR analysis of *Pro35S:CaGIR1* Arabidopsis plants. The *AtActin8* (*AtACT8*) gene was used as an internal control. (b) Seed germination of *Pro35S:CaGIR1* and wild-type (WT) plants in responses to ABA. Seeds were germinated on 0.5× MS agar plates containing various concentrations of ABA. The number of seeds with radicle emergence was recorded for 6 days after cold stratification. (c, d) Cotyledon greening of *Pro35S:CaGIR1* and WT plants on 0.5× MS agar plates containing 0.5 μM ABA. Representative images were taken 7 days after plating (c), and at the same time, the number of seedlings with green cotyledons was recorded (d). (e, f) Seedling growth of *Pro35S:CaGIR1* and WT plants in response to ABA. Seeds were germinated and grown vertically on 0.5× MS agar plates containing various concentrations of ABA. At 9 days after plating, representative images were taken (e) and root lengths of each plant were measured (f). (g, h) Post-germinative growth of *Pro35S:CaGIR1* and WT plants. Seeds of both plant lines were germinated on 0.5× MS agar plates for 2 days and then transferred to 0.5× MS medium containing 0, 10, and 20 μM ABA. After 5 days, representative images were taken (g), and root lengths were measured (h). All data represent the mean ± standard error of three independent experiments, each evaluating 36 seeds. Asterisks indicate significant differences between *Pro35S:CaGIR1* and WT plants (Student's *t*-test; *$P < 0.05$).

Table 1. List of primers used in this study.

Primer name	Primer sequence (5'-3')
For cloning	
CaGIR1-CDSF	Forward: ATGGCCTTAGAACAACTTTTTCAA
CaGIR1-CDSR	Reverse: TTATAGTTGCCTTCTTAACCTTGGA
CaGIR1-noSTF	Forward: AGTCCAAGGTTAAGAAGGCAACTAAAGGGCGAATTCG
CaGIR1-noSTR	Reverse: CGAATTCGCCCTTTAGTTGCCTTCTTAACCTTGGACT
CaGIR1 C59S/H61Y F	Forward: GCAAGGCCAGCAAAAGAGATATCCGCTTAACGTAACAACAGG ATCA
CaGIR1 C59S/H61Y R	Reverse: TGATCCTGTTGTTACGTTAAGCGGATATCTCTTTTGCTGGCCT TGC
CaGIR1-VIGS-F	Forward: TCTAGAATGGCCTTAGAACAACTTTT
CaGIR1-VIGS-R	Reverse: CTCGAGATTTTGGTTTCTGGTTATAG
For qRT-PCR analysis	
CaGIR1-RTF	Forward: AAGCAACAAAATCATCTGAAGGCA
CaGIR1-RTR	Reverse: TACTGCTGGCTGCTAGATTATATGAGT
CaACT1	Forward: GACGTGACCTAACTGATAACCTGAT
(CA12g08730)	Reverse: CTCTCAGCACCAATGGTAATAACTT
AtActin8	Forward: CAACTATGTTCTCAGGTATTGCAGA
(At1g49240)	Reverse: GTCATGGAAACGATGTCTCTTTAGT
NCED3	Forward: ACATGGAAATCGGAGTTACAGATAG
(At3g14440)	Reverse: AGAAACAACAAACAAGAAACAGAGC
RAB18	Forward: GGAAGAAGGGAATAACACAAAAGAT
(At5g66400)	Reverse: GCGTTACAAACCCTCATTATTTTTA
RD29B	Forward: GTTGAAGAGTCTCCACAATCACTTG
(At5g52300)	Reverse: ATACAAATCCCCAAACTGAATAACA
RD29B	Forward: GTTGAAGAGTCTCCACAATCACTTG
(At5g52300)	Reverse: ATACAAATCCCCAAACTGAATAACA
HAB1	Forward: TAGAAAATGCTGGAGGCAAAGTT
(At1g72770)	Reverse: TCAGGTTCTGGTCTTGAACTTTCTTT
